# Supplementary material for: TORC1 Signaling Controls the Stability and Function of α-Arrestins Aly1 and Aly2
Source: Biomolecules. 2022 Mar 31;12(4):533. doi: 10.3390/biom12040533 (PMC9031309; doi:10.3390/biom12040533)
Supplement: Supplementary file 1 [file biomolecules-12-00533-s001.zip › Supplemental KinDel manu_FIN.pdf]

**Supplemental Data File for TORC1 signaling controls the stability and function of α-arrestins Aly1 and Aly2**

**Supplemental Figure S1.** Serial dilution growth assays to further assess the KinDel library candidates

**Supplemental Figure S2.** Assessing *TEF1pr*-GFP abundance in KinDel gene deletion candidates

**Supplemental Figure S3**. Fluorescence distributions for Aly1- or Aly2-GFP in *sit4*Δ *npr1*Δ replicate experiments

**Supplemental Figure S4**. NBD2* as a control for MG132 proteasome degradation assays

**Supplemental Figure S5.** Loss of E3 ubiquitin ligases does not increase Aly protein abundance

**Supplemental Figure S6**. Impaired vacuole protease function increases $Aly^{PPXYless}$ protein abundance

**Supplemental Table S1.** Yeast strains used in this study.

**Supplemental Table S2.** Yeast plasmids used in this study.

**Supplemental Tables 3–4** are provided as separate Excel files

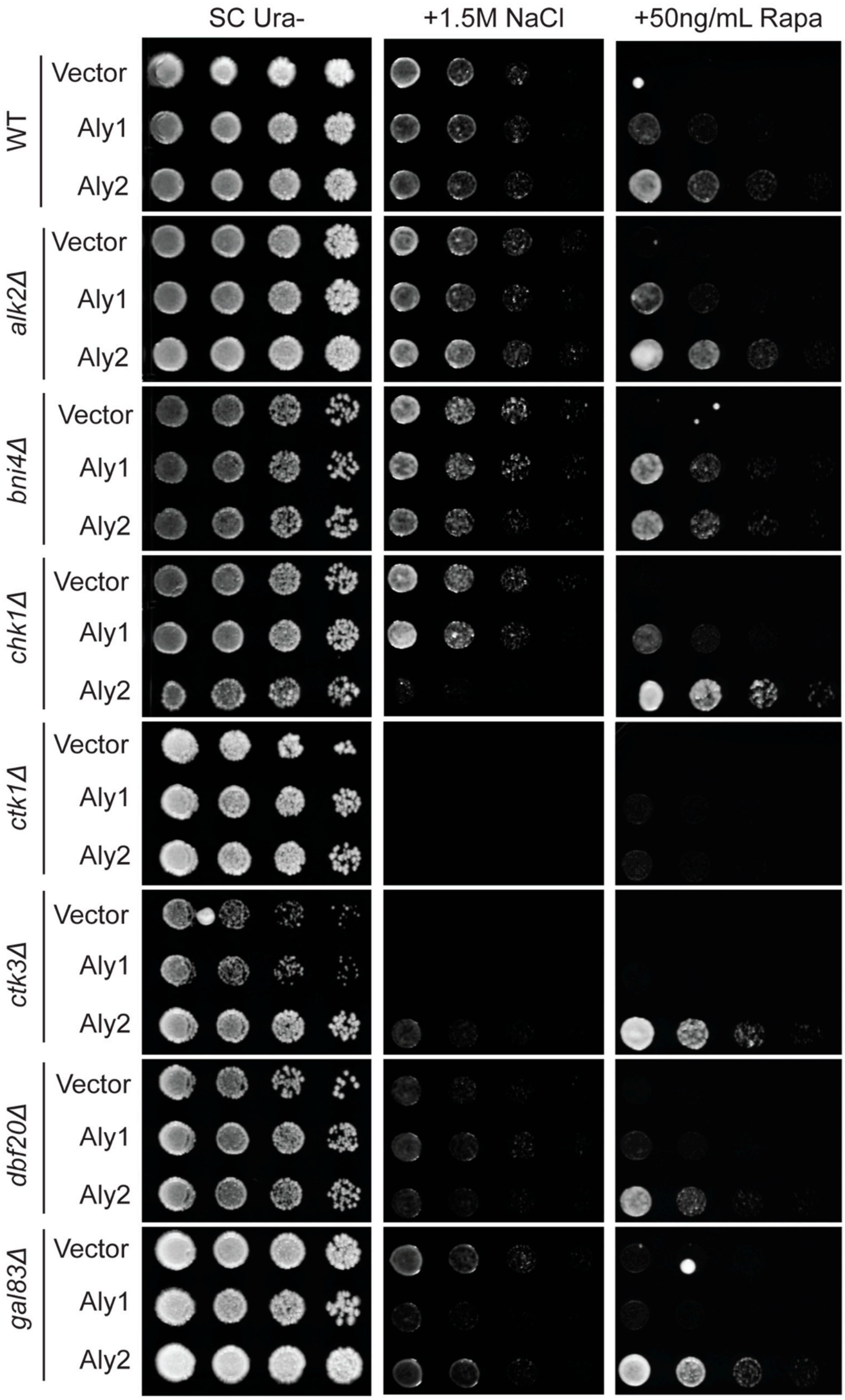
SC Ura-
+1.5M NaCl
+50ng/mL Rapa
WT
alk2Δ
bni4Δ
chk1Δ
ctk1Δ
ctk3Δ
dbf20Δ
gal83Δ
Vector
Aly1
Aly2

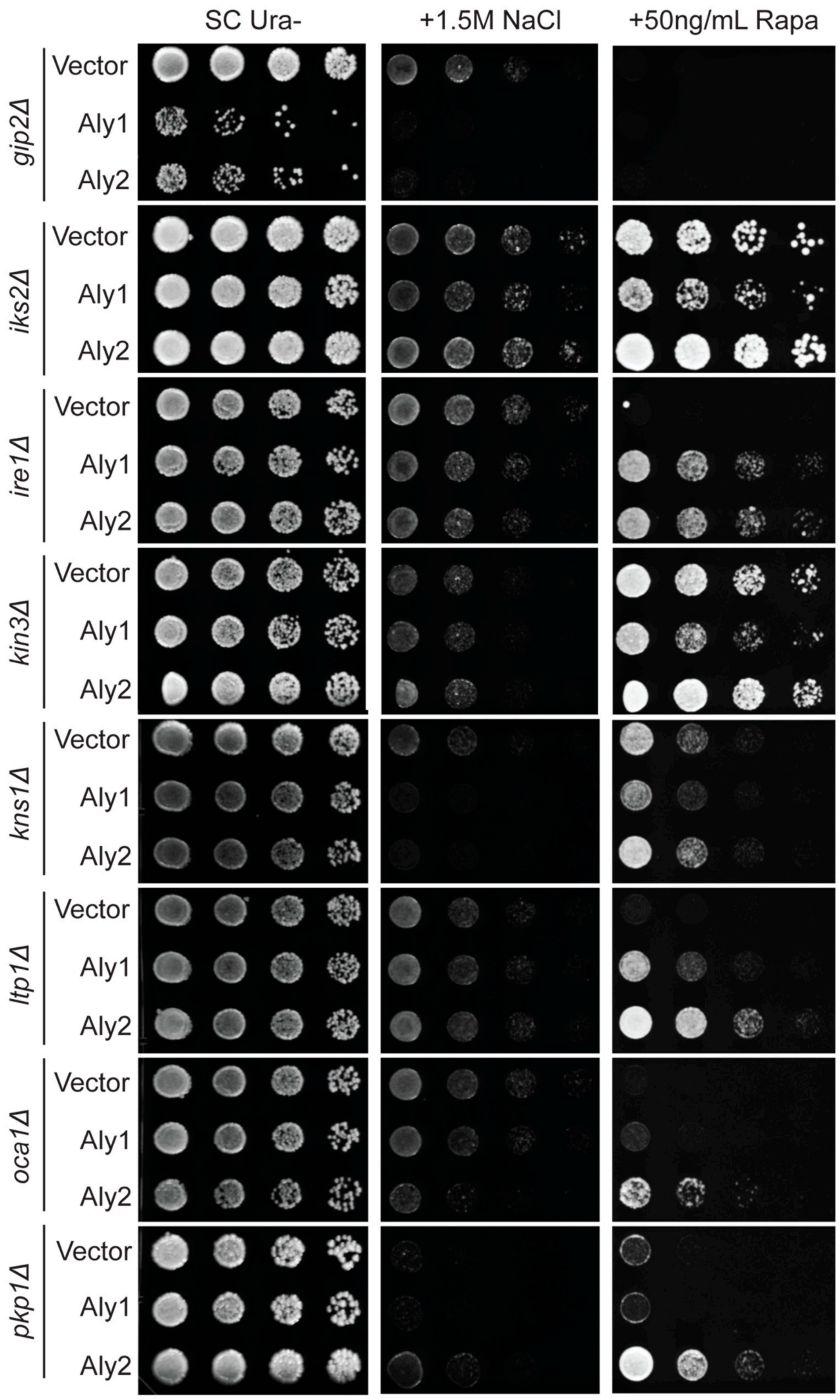
SC Ura-
+1.5M NaCl
+50ng/mL Rapa
gip2Δ
iks2Δ
ire1Δ
kin3Δ
kns1Δ
ltp1Δ
oca1Δ
pkp1Δ
Vector
Aly1
Aly2

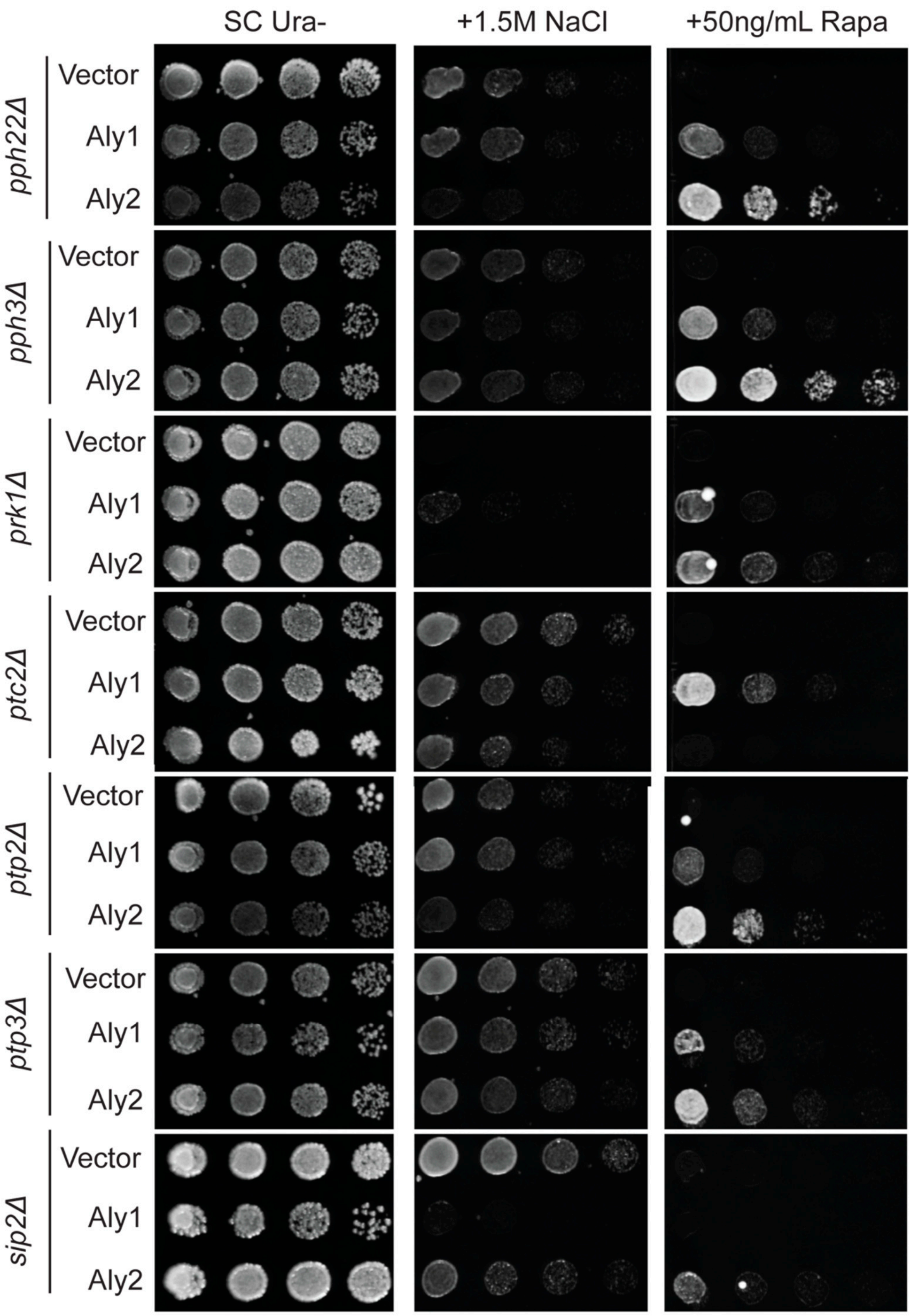
SC Ura-
+1.5M NaCl
+50ng/mL Rapa
pph22Δ
Vector
Aly1
Aly2
pph3Δ
Vector
Aly1
Aly2
prk1Δ
Vector
Aly1
Aly2
ptc2Δ
Vector
Aly1
Aly2
ptp2Δ
Vector
Aly1
Aly2
ptp3Δ
Vector
Aly1
Aly2
sip2Δ
Vector
Aly1
Aly2

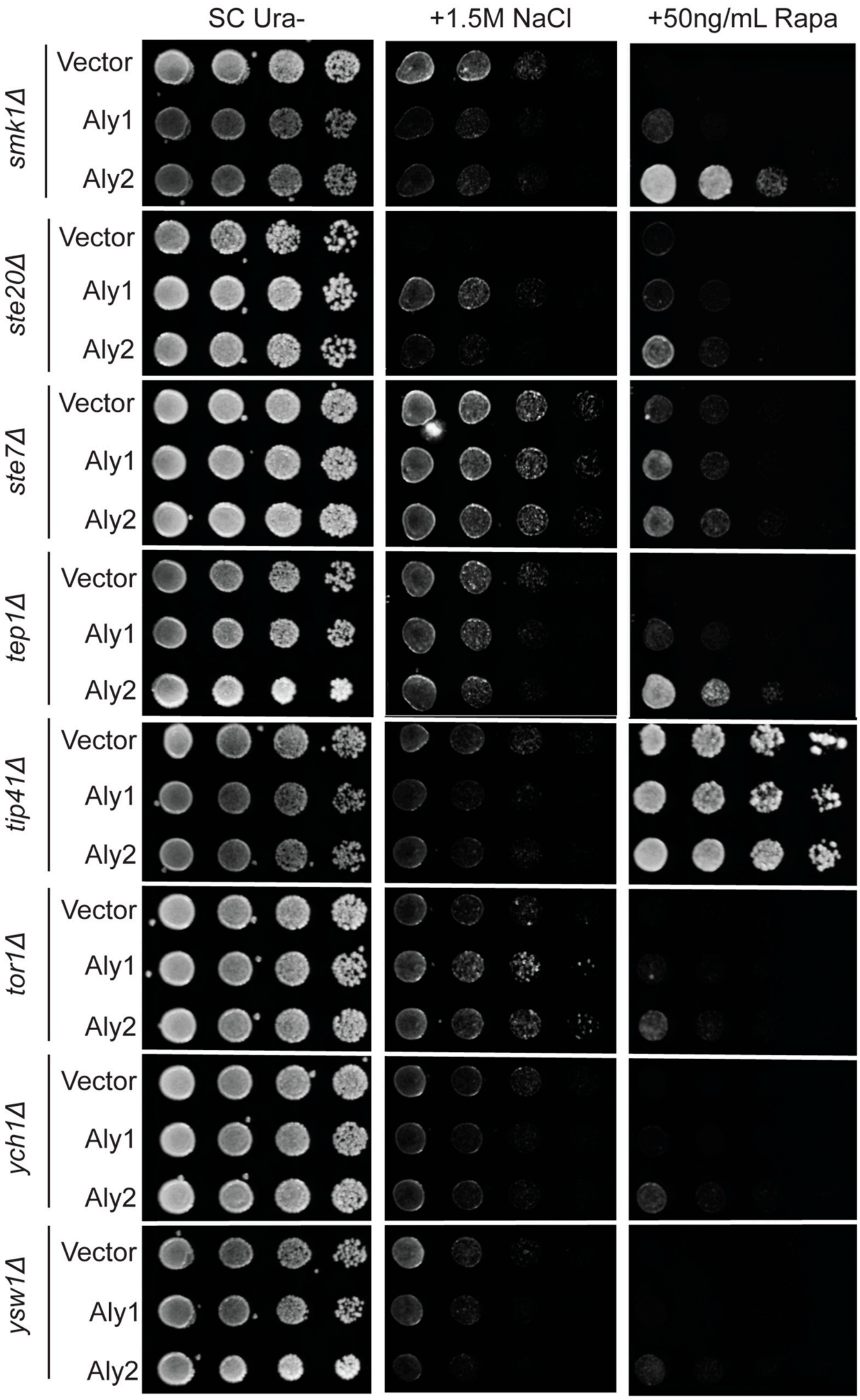
SC Ura-
+1.5M NaCl
+50ng/mL Rapa
smk1Δ
ste20Δ
ste7Δ
tep1Δ
tip41Δ
tor1Δ
ych1Δ
ysw1Δ
Vector
Aly1
Aly2

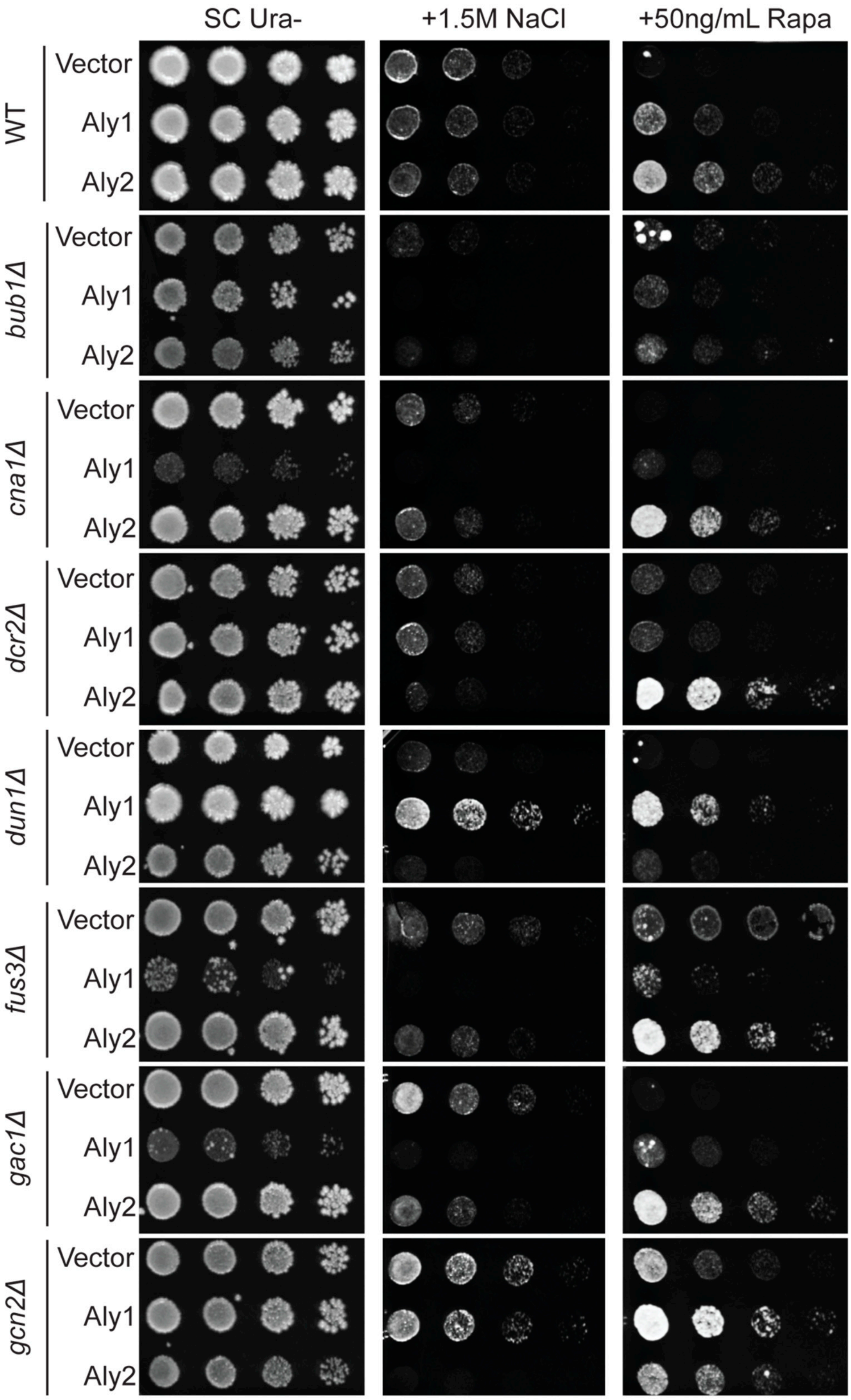

SC Ura-
+1.5M NaCl
+50ng/mL Rapa
WT
Vector
Aly1
Aly2
bub1Δ
cna1Δ
dcr2Δ
dun1Δ
fus3Δ
gac1Δ
gcn2Δ

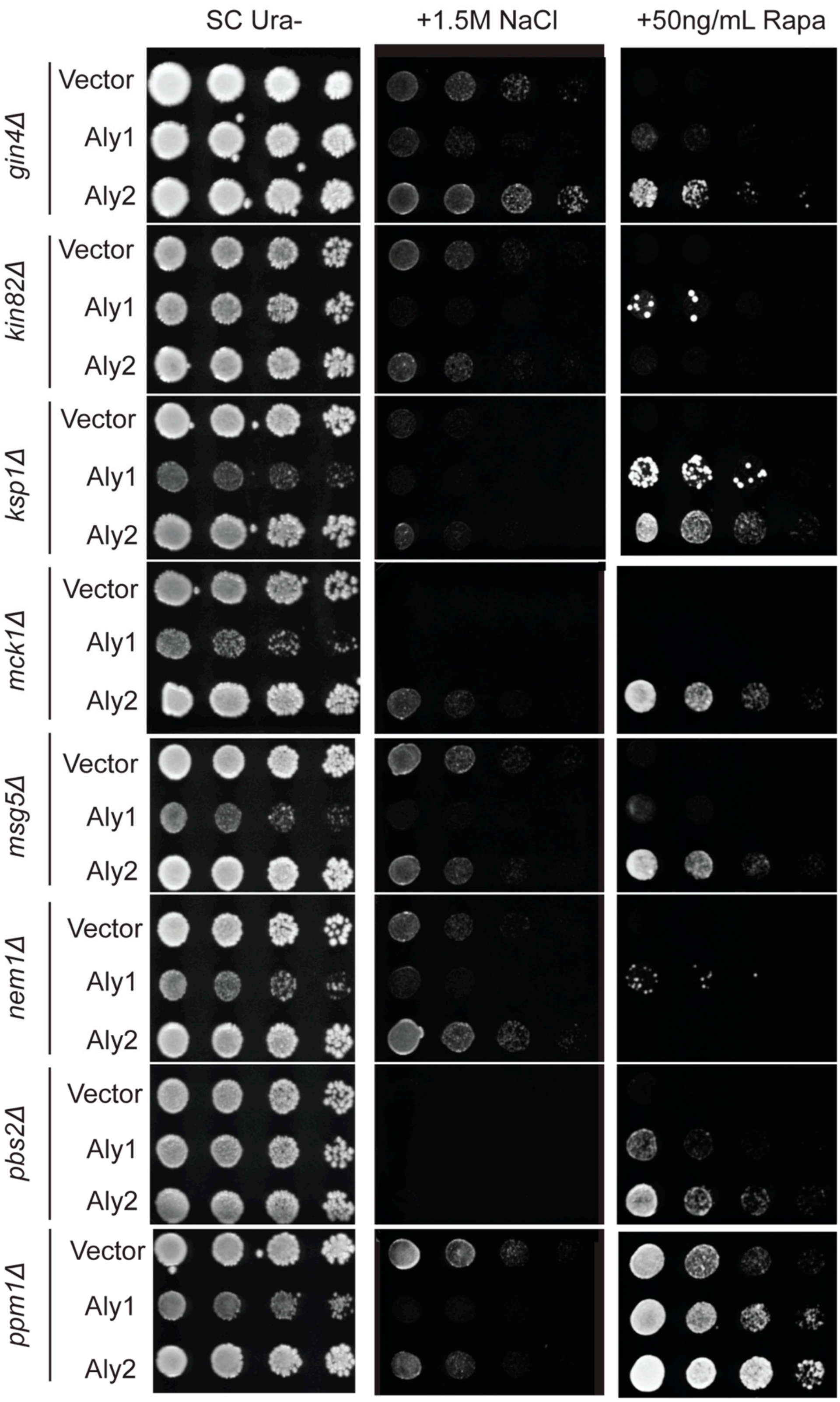
SC Ura-
+1.5M NaCl
+50ng/mL Rapa
gin4Δ
kin82Δ
ksp1Δ
mck1Δ
msg5Δ
nem1Δ
pbs2Δ
ppm1Δ
Vector
Aly1
Aly2

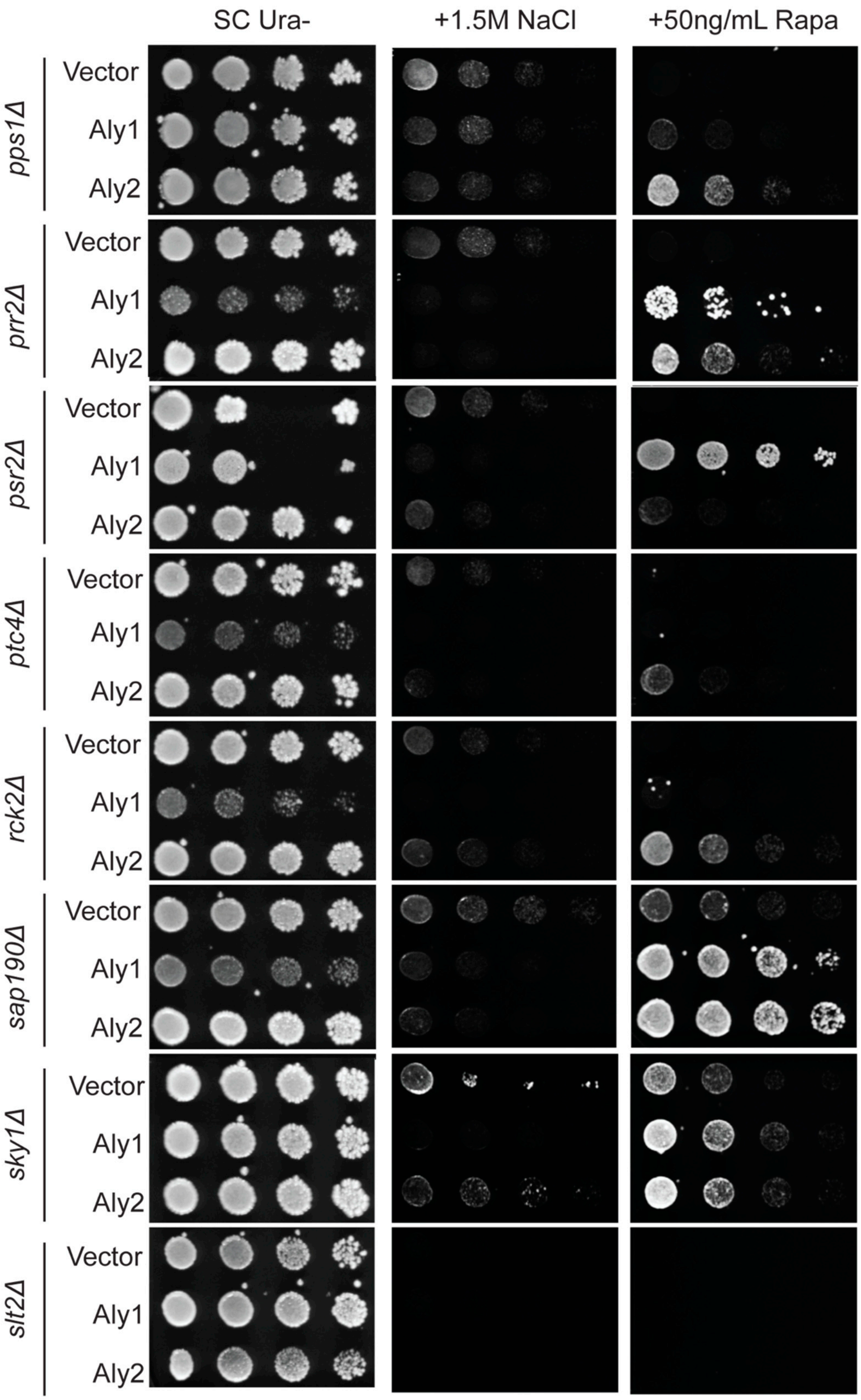
SC Ura-
+1.5M NaCl
+50ng/mL Rapa
pps1Δ
prr2Δ
psr2Δ
ptc4Δ
rck2Δ
sap190Δ
sky1Δ
slt2Δ
Vector
Aly1
Aly2

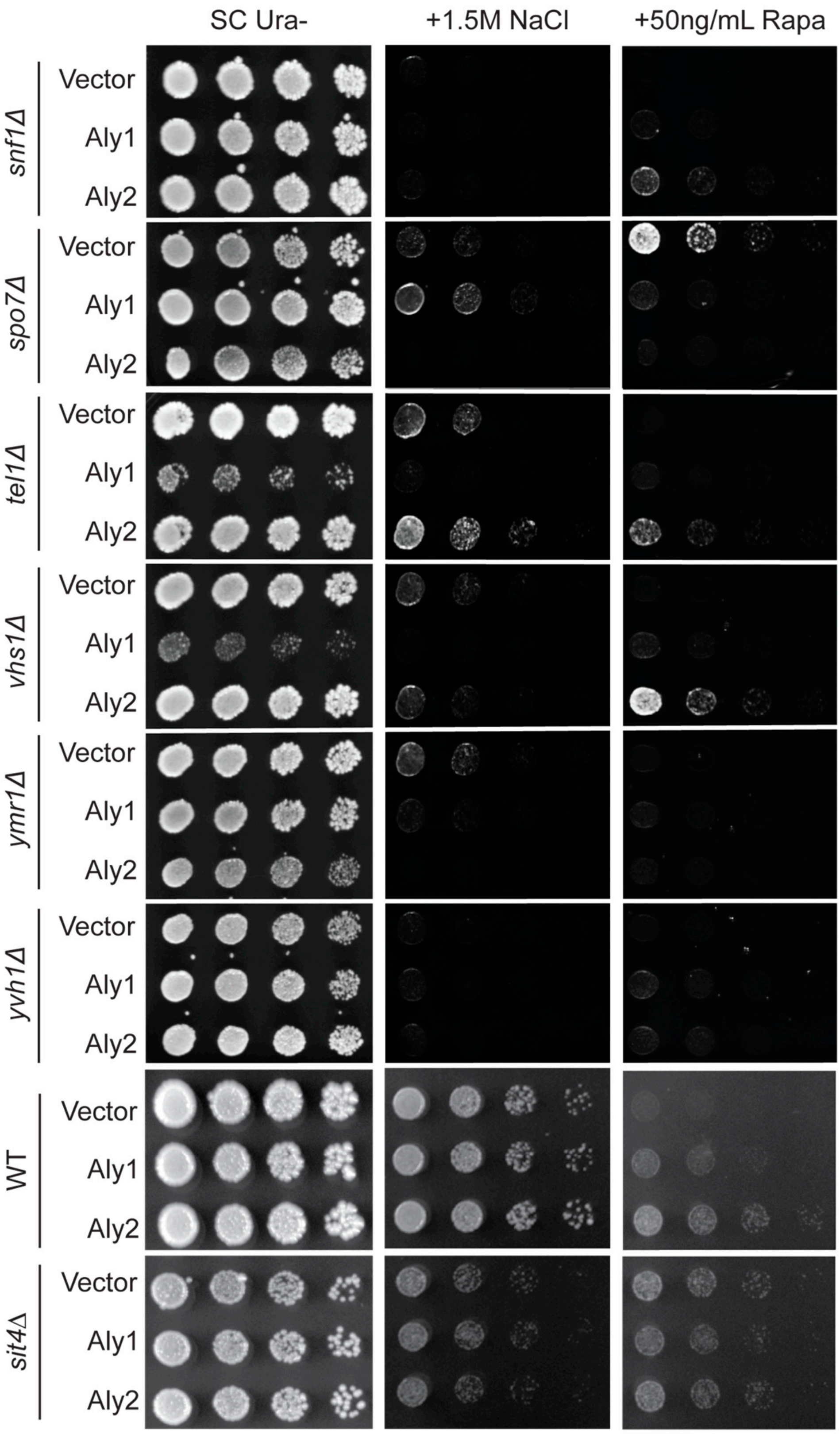
SC Ura-
+1.5M NaCl
+50ng/mL Rapa
snf1Δ
spo7Δ
tel1Δ
vhs1Δ
ymr1Δ
yvh1Δ
WT
sit4Δ
Vector
Aly1
Aly2

**Supplemental Figure S1. Serial dilution growth assays to further assess the KinDel library candidates**. a) Growth of serial dilutions of WT cells containing the indicated pRS426-derived plasmids expressing either nothing (vector) or the indicated $\alpha$-arrestin on SC medium lacking uracil and containing 1.5M NaCl or 50ng/mL rapamycin. These data represent a secondary screen of gene deletion candidates based on the initial high-content KinDel library screen. 60 candidates were tested in this way. It should be noted that some of the phenotypes for these assays are more severe than the phenotypes observed in subsequent growth assays (see Figure 2B) as the cells used to initiate these growth assays were older transformants than those used elsewhere.

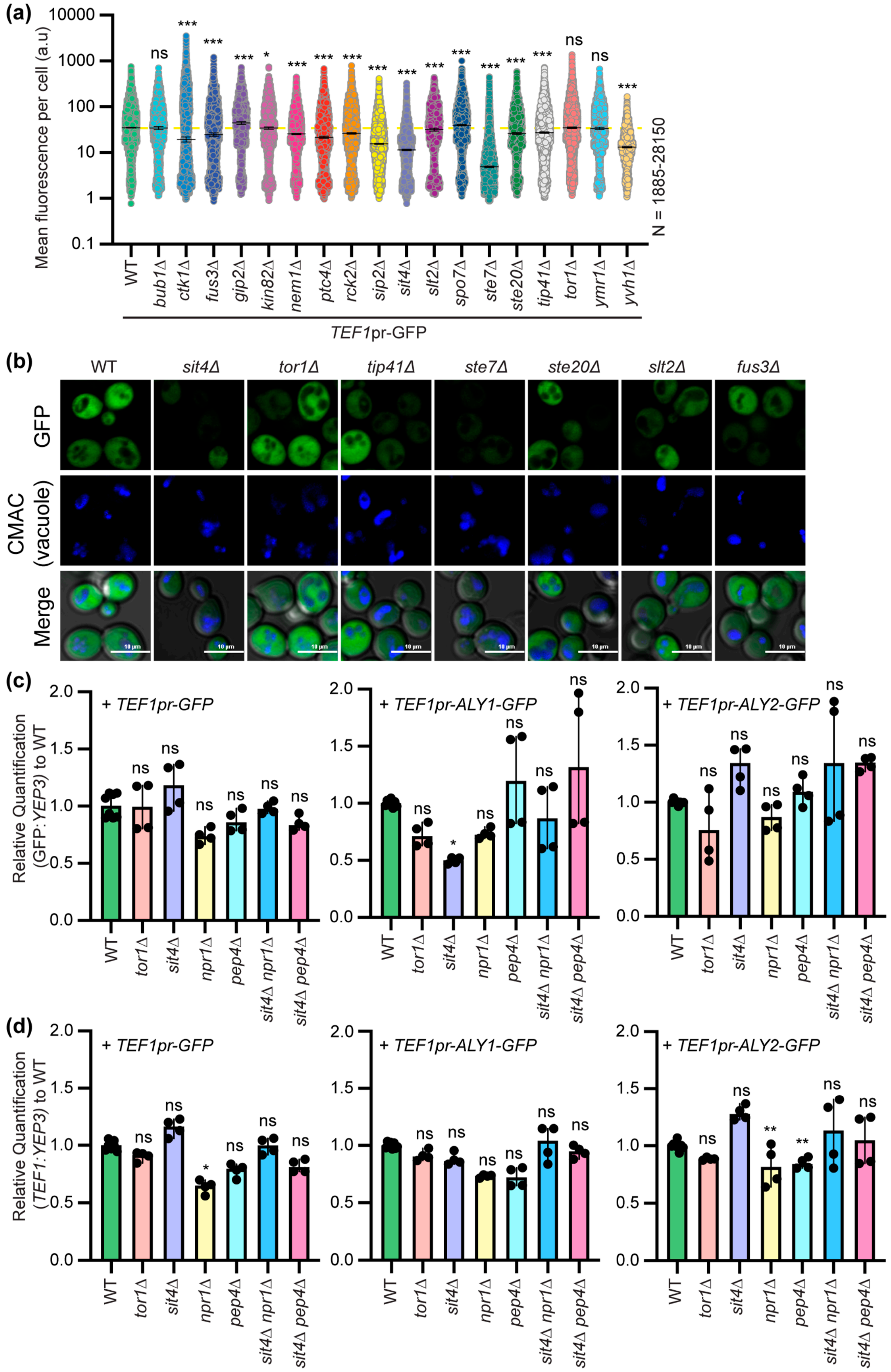
(a)
Mean fluorescence per cell (a.u)
10000
1000
100
10
1
0.1
ns
***
*
N = 1885-28150
WT
bub1Δ
ctk1Δ
fus3Δ
gip2Δ
kin82Δ
nem1Δ
ptc4Δ
rck2Δ
sip2Δ
sit4Δ
slt2Δ
spo7Δ
ste7Δ
ste20Δ
tip41Δ
tor1Δ
ymr1Δ
yvh1Δ
TEF1pr-GFP
(b)
WT
sit4Δ
tor1Δ
tip41Δ
ste7Δ
ste20Δ
slt2Δ
fus3Δ
GFP
CMAC (vacuole)
Merge
(c)
Relative Quantification (GFP:YEP3) to WT
+ TEF1pr-GFP
+ TEF1pr-ALY1-GFP
+ TEF1pr-ALY2-GFP
WT
tor1Δ
sit4Δ
npr1Δ
pep4Δ
sit4Δ npr1Δ
sit4Δ pep4Δ
(d)
Relative Quantification (TEF1:YEP3) to WT
+ TEF1pr-GFP
+ TEF1pr-ALY1-GFP
+ TEF1pr-ALY2-GFP
**

**Supplemental Figure S2. Assessing *TEF1pr*-GFP abundance in KinDel gene deletion candidates**. a) Cells expressing the pRS415-*TEF1pr*-GFP were imaged by high-content confocal microscopy and the whole cell fluorescence was quantified using Nikon.*ai* software. The mean fluorescence intensity from whole cell measurements (in arbitrary units, au) is plotted for each cell as a circle. The median fluorescence intensity is shown as a black line for each group and the error bars represent the 95% confidence interval. A yellow dashed line represents the median fluorescence intensity for GFP expressed in WT cells. Kruskal-Wallis statistical analysis with Dunn's post hoc test was performed to compare the GFP fluorescence distributions to that of WT cells. In black asterisks, statistical comparisons are made to GFP in WT cells. (ns = not significant; one symbol has a p-value <0.05; three symbols have a p-value <0.0005). b) A subset of the fluorescent microscopy images acquired for the data presented in (a) are shown. CMAC is used to stain the vacuoles (shown in blue). While in some instances the abundance changes for free GFP mirrors that of the Alys fused to GFP, the fluorescence is much higher for free GFP than any of the Aly-GFP fusions (~ an order of magnitude higher for GFP than the Alys). c-d) Relative quantifications of qRT-PCR analyses of (c) GFP or (d) chromosomal *TEF1* transcripts to the *YEP3* control transcripts are presented for four replicate experiments. Transcript abundances in cells bearing gene deletions and expressing the indicated *TEF1pr*-X-GFP plasmid are presented relative to the transcript abundance in the WT controls. Kruskal-Wallis statistical analysis with Dunn's post hoc test was performed to compare the transcript abundances to the cognate WT (ns = not significant; * = p-value<0.05; ** = p-value <0.005)

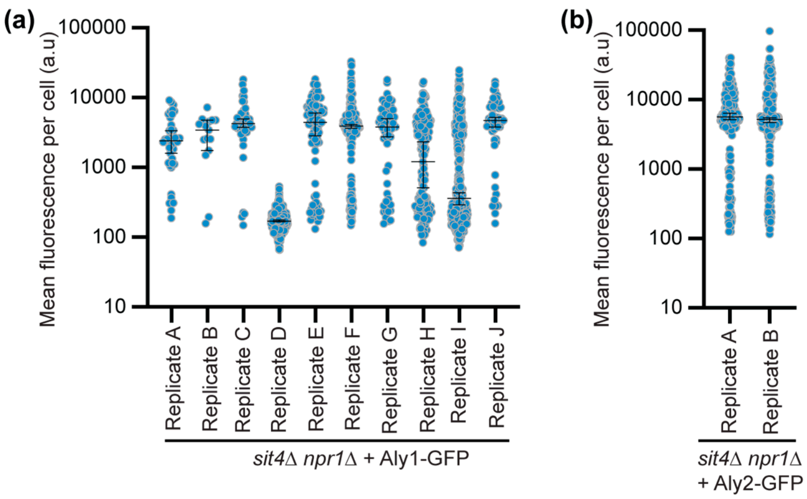


**Supplemental Figure S3. Fluorescence distributions for Aly1- or Aly2-GFP in *sit4Δ npr1Δ* replicate experiments**. Whole cell fluorescence was quantified for *sit4Δ npr1Δ* cells expressing (a) Aly1-GFP or (b) Aly2-GFP across the biological replicate experiments indicated (A-J for Aly1 and A-B for Aly2) using Nikon.*ai* software. The mean fluorescence intensity from whole cell measurements (in arbitrary units, au) is plotted for each cell as a circle. The median fluorescence intensity is shown as a black line for each group and the error bars represent the 95% confidence interval. This figure demonstrates the high degree of variability in the Aly1-GFP abundance across 10 replicate experiments in the *sit4Δ npr1Δ* background.

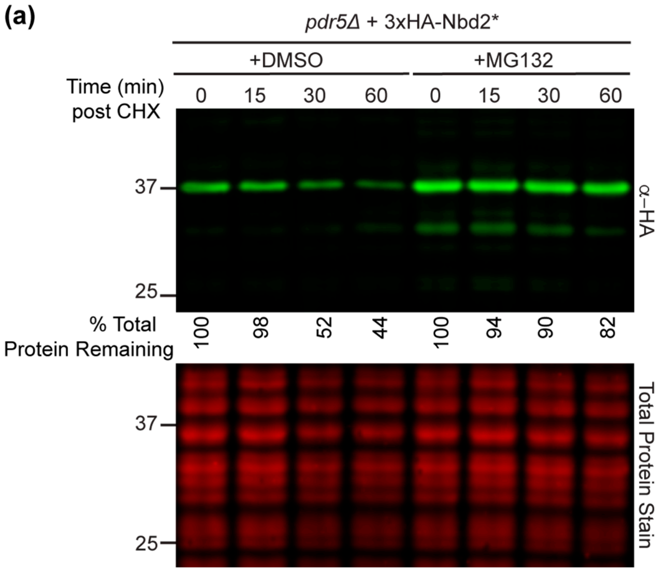


**Supplemental Figure S4. NBD2* as a control for MG132 proteasome degradation assays**. a) Whole-cell extracts from cells expressing 3xHA-Nbd2* were treated with either vehicle control (DMSO) or MG132 for 60 minutes and then cycloheximide (CHX) for the time indicated (hours) and were resolved by SDS-PAGE. Anti-HA antibody was used to detect tagged Nbd2* and REVERT total protein stain was used as a loading control. Blots shown are one representative of 2 replicate experiments. Molecular weights are shown on the left side in kDa. For each lane, the pixel intensities for the HA-detected band and the loading control were measured using Image J. A correction factor based on the loading control was applied to each pixel intensity measurement. The t=0 point was set to 100% and the % of protein remaining is indicated for each time point. Based on these assays we find significant stabilization of Nbd2*. Nbd2* serves as a positive control for our MG132-dependent proteasome degradation assays with Aly-GFP (Figure 8).

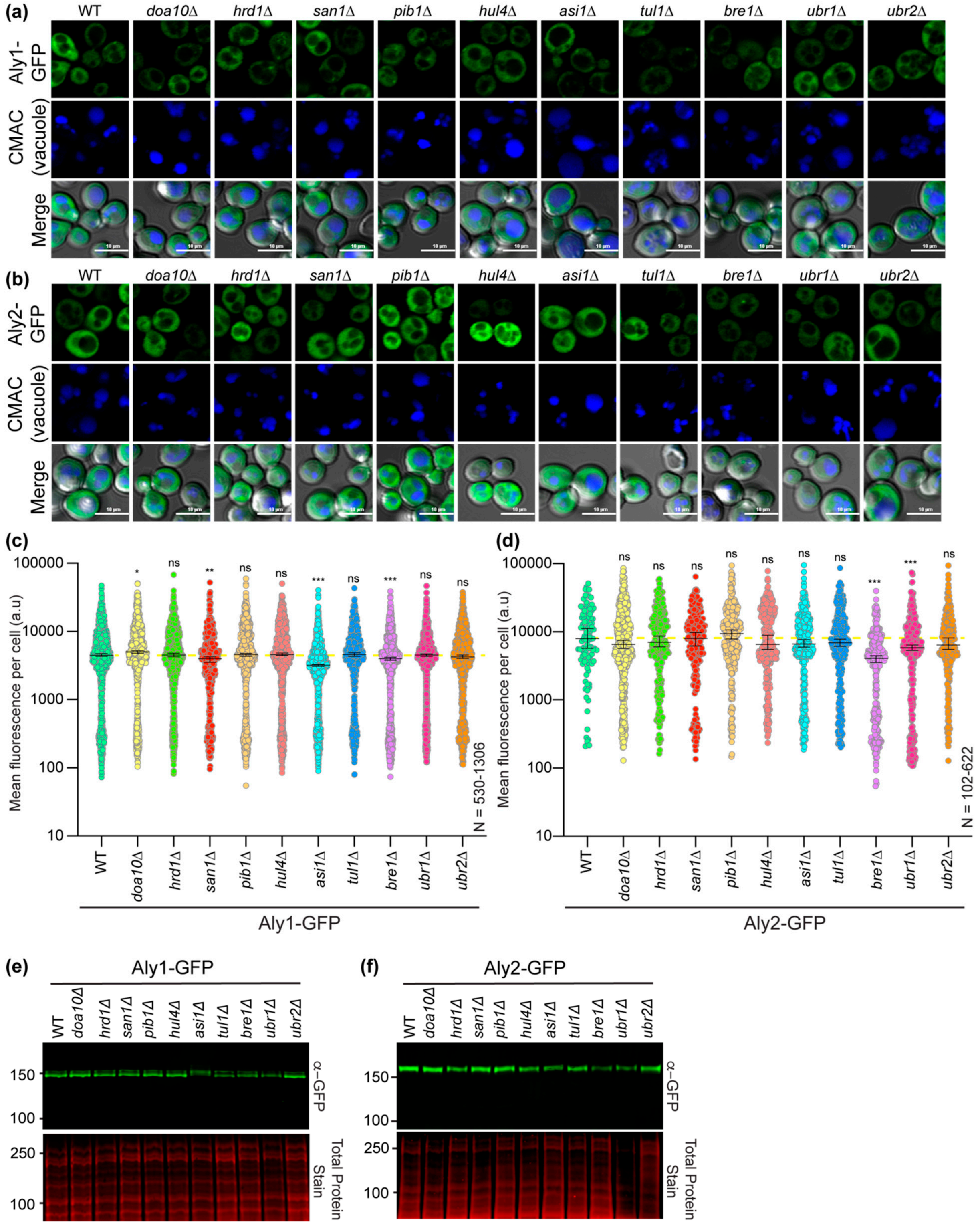


**Supplemental Figure S5. Loss of E3 ubiquitin ligases does not increase Aly protein abundance.** a) Aly1-GFP or c) Aly2-GFP was expressed from the *TEF1pr* in either WT cells or those lacking the gene indicated and imaged by high-content fluorescence microscopy. CMAC is used to stain the vacuoles (shown in blue). All images are shown as equally adjusted from a single experiment. c or d) The whole cell fluorescence of cells imaged in a) or b), respectively,

was determined using Nikon AI software and the fluorescence for each cell is plotted as a circle. The median fluorescence intensity is shown as a black line for each group and the error bars represent the 95% confidence interval. A yellow dashed line represents the median fluorescence intensity for Aly1 or Aly2 expressed in WT cells in panels b and d, respectively. Kruskal-Wallis statistical analysis with Dunn's post hoc test was performed to compare the fluorescence distributions to the cognate WT. In black asterisks, statistical comparisons are made to Aly1 or Aly2 in WT cells for panels c and d, respectively. e) and f) Whole-cell extracts from the cells as imaged in a) and b), respectively, were analyzed by SDS-PAGE and detected using an anti-GFP antibody. The REVERT total protein stain of the membrane is shown as a loading control. Molecular weights are shown on the left side in kDa.

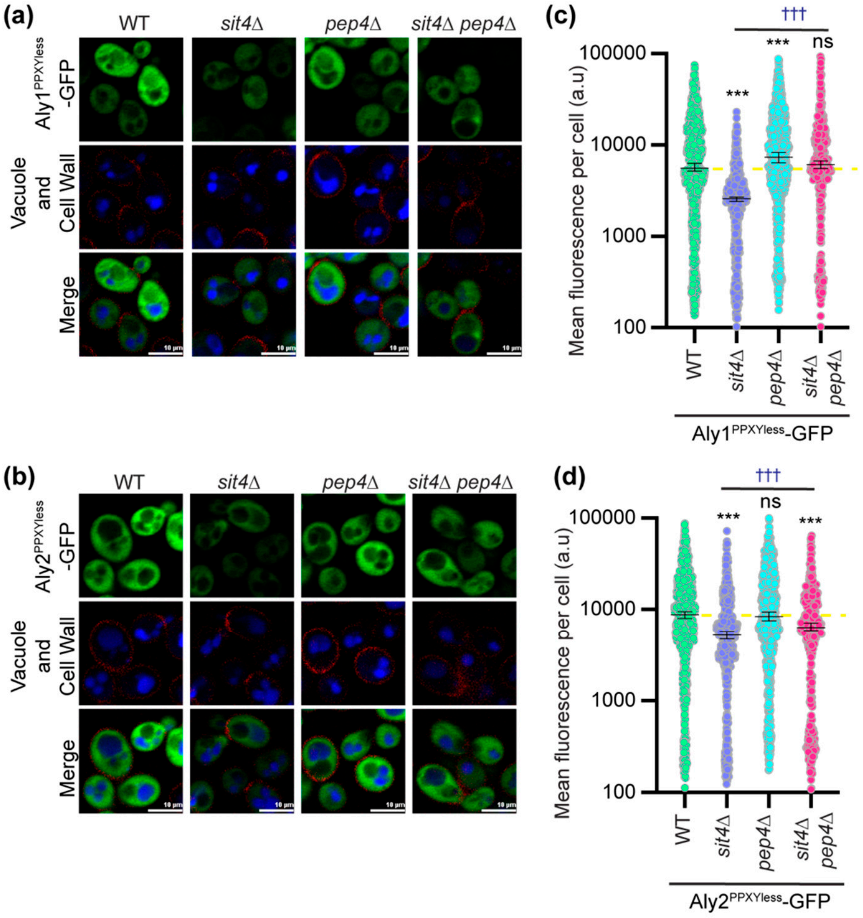


**Supplemental Figure S6. Impaired vacuole protease function increases Aly$^{PPXYless}$ protein abundance**. a) Aly1$^{PPXYless}$-GFP or b) Aly2$^{PPXYless}$-GFP was expressed from the *TEF1pr* in either WT cells or those lacking the gene indicated and imaged by high-content fluorescence microscopy. CMAC is used to stain the vacuoles (shown in blue) and trypan blue (shown in red) is used to mark the cell wall. c or d) The whole cell fluorescence of cells imaged in a) or b), respectively, was determined using Nikon AI software and the fluorescence for each cell is plotted as a circle. The median fluorescence intensity is shown as a black line for each group and the error bars represent the 95% confidence interval. A yellow dashed line represents the median fluorescence intensity for Aly1 or Aly2 expressed in WT cells in panels g and h, respectively. Kruskal-Wallis statistical analysis with Dunn's post hoc test was performed to compare the fluorescence distributions to the cognate WT. In black asterisks statistical comparisons are made to Aly1$^{PPXYless}$ or Aly2$^{PPXYless}$ in WT cells for panels c and d, respectively. In blue daggers, statistical comparisons are made to Aly1$^{PPXYless}$ or Aly2$^{PPXYless}$ in *sit4*Δ cells for panels c and d, respectively. (ns = not significant; three symbols = p-value <0.0005).

**Supplemental Table S1. Yeast strains used in this study.**

| Strain | Genotype | Source/Reference |
|---|---|---|
| BY4741 | *MAT* **a** *his3Δ1 leu2Δ0 ura3Δ0 met15Δ0* | [56] |
| *bub1Δ* | *MAT* **a** *bub2*Δ::KanMX *his3Δ1 leu2Δ0 ura3Δ0 met15Δ0* | [56] |
| *ctk1Δ* | *MAT* **a** *ctk1*Δ::KanMX *his3Δ1 leu2Δ0 ura3Δ0 met15Δ0* | [56] |
| *fus3Δ* | *MAT* **a** *fus3*Δ::KanMX *his3Δ1 leu2Δ0 ura3Δ0 met15Δ0* | [56] |
| *gip2Δ* | *MAT* **a** *gip2*Δ::KanMX *his3Δ1 leu2Δ0 ura3Δ0 met15Δ0* | [56] |
| *kin82Δ* | *MAT* **a** *kin82*Δ::KanMX *his3Δ1 leu2Δ0 ura3Δ0 met15Δ0* | [56] |
| *nem1Δ* | *MAT* **a** *nem1*Δ::KanMX *his3Δ1 leu2Δ0 ura3Δ0 met15Δ0* | [56] |
| *ptc4Δ* | *MAT* **a** *ptc4*Δ::KanMX *his3Δ1 leu2Δ0 ura3Δ0 met15Δ0* | [56] |
| *rck2Δ* | *MAT* **a** *rck2*Δ::KanMX *his3Δ1 leu2Δ0 ura3Δ0 met15Δ0* | [56] |
| *sip2Δ* | *MAT* **a** *sip2*Δ::KanMX *his3Δ1 leu2Δ0 ura3Δ0 met15Δ0* | [56] |
| *sit4Δ* | *MAT* **a** *sit4*Δ::KanMX *his3Δ1 leu2Δ0 ura3Δ0 met15Δ0* | [56] |
| *slt2Δ* | *MAT* **a** *slt2*Δ::KanMX *his3Δ1 leu2Δ0 ura3Δ0 met15Δ0* | [56] |
| *spo7Δ* | *MAT* **a** *spo7*Δ::KanMX *his3Δ1 leu2Δ0 ura3Δ0 met15Δ0* | [56] |
| *ste20Δ* | *MAT* **a** *ste20*Δ::KanMX *his3Δ1 leu2Δ0 ura3Δ0 met15Δ0* | [56] |
| *ste7Δ* | *MAT* **a** *ste7*Δ::KanMX *his3Δ1 leu2Δ0 ura3Δ0 met15Δ0* | [56] |
| *tip41Δ* | *MAT* **a** *tip41*Δ::KanMX *his3Δ1 leu2Δ0 ura3Δ0 met15Δ0* | [56] |
| *tor1Δ* | *MAT* **a** *tor1*Δ::KanMX *his3Δ1 leu2Δ0 ura3Δ0 met15Δ0* | [56] |
| *ymr1Δ* | *MAT* **a** *ymr1*Δ::KanMX *his3Δ1 leu2Δ0 ura3Δ0 met15Δ0* | [56] |
| *yvh1Δ* | *MAT* **a** *yvh1*Δ::KanMX *his3Δ1 leu2Δ0 ura3Δ0 met15Δ0* | [56] |
| *npr1Δ* | *MAT* **a** *npr1*Δ::KanMX *his3Δ1 leu2Δ0 ura3Δ0 met15Δ0* | [56] |

| Strain | Genotype | Source/Reference |
|---|---|---|
| *sit4*Δ *npr1*Δ | *MAT* **a** *sit4*Δ::KanMX *npr1*Δ::HphMX *his3Δ1 leu2Δ0 ura3Δ0 met15Δ0* | This study<br>Using the *sit4*Δ::KanMX strain from the deletion collection, we PCR amplified the HphMX cassette [57] using primers with homology to the regions up- and downstream of the Npr1 coding sequence. We then validated that Npr1 was deleted using a PCR-based approach. |
| BY4742 | *MAT α his3Δ1 leu2Δ0 ura3Δ0 lys2Δ0* | [56] |
| *pdr5*Δ | *MAT α pdr5*Δ::KanMX *his3Δ1 leu2Δ0 ura3Δ0 lys2Δ0* | [56] |
| *pep4*Δ | *MAT **a** pep4*Δ::KanMX *his3Δ1 leu2Δ0 ura3Δ0 met15Δ0* | This study<br>We PCR amplified the KanMX cassette [58] using primers with homology to the regions up- and downstream of the Pep4 coding sequence. We then validated that Pep4 was deleted using a PCR-based approach. |
| *sit4*Δ *pdr5*Δ | *MAT* **a** *sit4*Δ::KanMX *pdr5*Δ::HphMX *his3Δ1 leu2Δ0 ura3Δ0 met15Δ0* | This study<br>Using the *sit4*Δ::KanMX strain from the deletion collection, we PCR amplified the HphMX cassette [57] using primers with homology to the regions up- and downstream of the Pdr5 coding sequence. We then validated that Pdr5 was deleted using a PCR-based approach. |
| *sit4*Δ *pep4*Δ | *MAT* **a** *sit4*Δ::KanMX *pep4*Δ::HphMX *his3Δ1 leu2Δ0 ura3Δ0 met15Δ0* | This study<br>Using the *sit4*Δ::KanMX strain from the deletion collection, we PCR amplified the HphMX cassette [57] using primers with homology to the regions up- and downstream of the Pep4 coding sequence. We then validated that Pep4 was deleted using a PCR-based approach. |
| *aly1*Δ *aly2*Δ (aka D2-6a) | *MAT* **a** *aly1*Δ::KanMX *aly2*Δ::KanMX *his3Δ1 leu2Δ0 ura3Δ0 met15Δ0 lys2Δ0* | [5] |
| *9Arr*Δ (EN60) | *ecm21Δ::KanMX csr2Δ::KanMX bsd2Δ rog3Δ::NatMX rod1Δ ygr068cΔ aly1Δ aly2Δ ylr392cΔ::HIS3 his3Δ0 ura3Δ0 leu2Δ0* | [4] |

**Supplemental Table 2. Plasmids used in this study.**

| Plasmid | Genotype | Description (Reference) |
|---|---|---|
| pRS415 | *TEF1prom*, CEN, *LEU2* | [60] |
| pRS415-*TEF1pr*-GFP | *TEF1prom-GFP*, CEN, *LEU2* | [61] |
| pRS415-Aly1-GFP | *TEF1prom-ALY1*, CEN, *LEU2* | This study. The coding region of Aly1 lacking its stop codon was PCR amplified using pRS426-Aly1 as a template and primers with *Spe*I and *Pst*I restriction site adaptors. These were then subcloned into pRS415-*TEF1pr*-GFP at the *Spe*I and *Pst*I sites. |
| pRS415-Aly2-GFP | *TEF1prom- ALY2*-GFP, CEN, *LEU2* | This study. The coding region of Aly2 lacking its stop codon was PCR amplified using pRS426-Aly2 as a template and primers with *Spe*I and *Pst*I restriction site adaptors. These were then subcloned into pRS415-*TEF1pr*-GFP at the *Spe*I and *Pst*I sites. |
| pRS415-Aly1$^{PPxY\text{-}less}$-GFP | *TEF1prom-aly1*$^{PPxYless}$, CEN, *LEU2* | This study. The coding region of Aly1$^{PPxYless}$ lacking its stop codon was PCR amplified using pRS426-Aly1$^{PPxYless}$ as a template and primers with *Spe*I and *Pst*I restriction site adaptors. These were then subcloned into pRS415-*TEF1pr*-GFP at the *Spe*I and *Pst*I sites. |
| pRS415-Aly2$^{PPxY\text{-}less}$-GFP | *TEF1prom- aly2*$^{PPxYless}$ -GFP, CEN, *LEU2* | This study. The coding region of Aly2$^{PPxYless}$ lacking its stop codon was PCR amplified using pRS426-Aly2$^{PPxYless}$ as a template and primers with *Spe*I and *Pst*I restriction site adaptors. These were then subcloned into pRS415-*TEF1pr*-GFP at the *Spe*I and *Pst*I sites. |
| pRS415-Git1-GFP | *TEF1prom-GIT1*-GFP, 2μ, *LEU2* | [61] |
| pRS426 | 2μ, *URA3* | [62] |
| pRS426-Aly1 | *ALY1prom-ALY1*, 2μ, *URA3* | [5] |
| pRS426-Aly2 | *ALY2prom-ALY2*, 2μ, *URA3* | [5] |

| Plasmid | Genotype | Description (Reference) |
|---|---|---|
| pRS426-Aly1$^{PPxYless}$ | *ALY1prom-aly1$^{PPxYless}$*, 2μ, *URA3* | [10] |
| pRS426-Aly2$^{PPxYless}$ | *ALY2prom-aly2$^{PPxYless}$*, 2μ, *URA3* | [10] |
| pCG04 | ste6–166NBD2–3HA, 2μ, *URA3* | [63] |
